# Overcoming Global Antifungal Challenges: Medical and Agricultural Aspects

**DOI:** 10.1021/acsbiomedchemau.5c00103

**Published:** 2025-07-02

**Authors:** László Galgóczy

**Affiliations:** Department of Biotechnology and Microbiology, Faculty of Science and Informatics, 37442 University of Szeged, Szeged 6726, Hungary

**Keywords:** human pathogenic fungus, phytopathogenic fungus, antifungal drug, fungicide, antifungal therapy, plant pathogenic fungi management, resistance, drug development

## Abstract

The prevalence of fungal infections and contamination has increased alarmingly over the past decade, posing a significant threat to human health and the food supply and negatively affecting welfare. This escalating concern is primarily attributed to the lack of safe, effective, and widely available antifungal agents; the increasing spread of (multi)­drug resistance to conventional antifungal treatments; and substantial epidemiological shifts in fungal pathogens. Decision-making bodies have recognized the urgency of this situation and prioritized efforts to address and mitigate the spread of drug-resistant fungal infections by developing and implementing innovative antifungal strategies, including using drug combinations, designing fundamentally new antifungal compounds with fungus-specific mechanisms of action and a minimal risk of resistance development, drug repurposing, and exploring alternative approaches, such as biomolecules, nanotechnology, and biological control. This review aims to provide a comprehensive overview of the current challenges associated with fungal infections in medicine and agriculture as well as the latest advancements and potential solutions.

## Graphical abstract

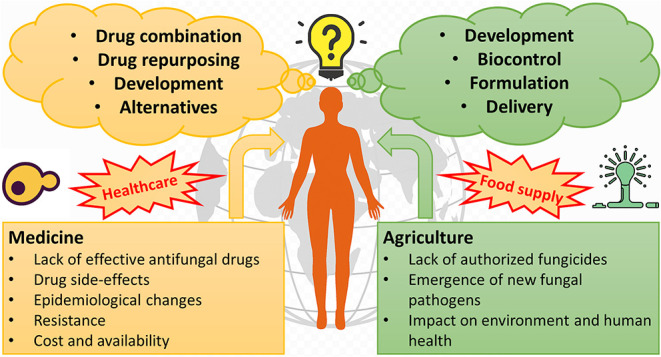

## 1 Introduction

The incidence of fungal infections and damage caused by fungi has recently reached alarming levels, and it has become evident that fundamentally different treatment and preventive strategies from those currently in use are necessary for both medicine and agriculture.^1^

Epidemiological survey data have revealed a significant increase in fatal human infections caused by drug-resistant fungi over the past 2 decades, mainly among immunosuppressed patients.^2,3^ The results from a 2020 survey suggested that of the approximately 150 million severe fungal infections that occur annually, around 1.7 million result in death,^2^ while a more recent study in 2024 estimated this number to be as high as 3.8 million.^3^ Although some authors argue that the mortality rate may be under- or overestimated due to irregular and unreliable epidemiological surveys, the evidence suggests that fatal fungal infections have doubled within just a few years.^4^ This is attributed to epidemiological changes,^5^ the increasing number of immunosuppressed patients, and the decreasing number of effective antifungal drugs.^6^ Fungi generally cause non-life-threatening but difficult-to-treat mycoses in immunocompetent individuals, mainly affecting the skin and mucosal surfaces. However, in immunosuppressed patients, these superficial infections may become invasive and involve the entire body, with a high rate of mortality.^7^ The lack of effective antifungal agents hampers the treatment of invasive fungal infections. Furthermore, those currently available may cause severe side effects in cases of prolonged therapy, permanently damaging organs of patients due to the similarities in cell structure, function, and metabolism to fungal cells.^6^ In clinical practice, the most critical challenge is the increasing resistance of fungi to one or more antifungal drugs with different mechanisms of action.^8^ Despite these troubling facts, this issue had received limited attention, prompting the scientific community to push for solutions at the policymaking level.^9^ To address this concern and raise public awareness, the World Health Organization (WHO) released its first statement on the issue of fungal infections in 2022, with the publication of the WHO fungal priority pathogens list (FPPL) to guide research, development, and public health action. This statement lists the 19 fungal pathogens posing the most significant public health risk and highlights research (Table 1), development, and action goals. It emphasizes the urgent need to prevent and control the spread of fungal infections and their resistance to antifungal drugs by introducing innovative strategies based on fundamentally different antifungal agents with novel fungus-specific mechanisms of action.^10,11^

**Table 1:** 19 Fungal Pathogens Posing the Most Significant Public Health Risk and Highlights Research^10^

critical group	high group	medium group
*Cryptococcus neoformans*	*Nakaseomyces glabrata* (formerly *Candida glabrata*)	*Scedosporium* spp.
	*Histoplasma* spp.	*Lomentospora prolificans*
*Candidozyma auris* (formerly *Candida auris*)	Eumycetoma causative agents	*Coccidioides* spp.
*Aspergillus fumigatus*	Mucorales	*Pichia kudriavzeveii* (formerly *Candida krusei*)
*Candida albicans*	*Fusarium* spp.	*Cryptococcus gattii*
	*Candida tropicalis*	*Talaromyces marneffei*
	*Candida parapsilosis*	*Pneumocystis jirovecii*
		*Paracoccidioides* spp.

Preventing and treating fungal infections poses a tremendous challenge not only for healthcare^12^ but also for agriculture. The amount of pre- (in-field) and postharvest (under storage) damage caused by plant pathogenic fungi has shown a steady global increase in recent years, resulting in losses of billions of dollars. Surveys have reported that 10–23% of annual crop yields are lost in the field, while an additional 10–20% is wasted due to fungal damage during storage. The economic losses pale in comparison with their impact on ensuring a food supply for the growing global population. If this loss is considered in relation to the five most important crops for human nutrition (i.e., rice, wheat, maize, soybean, and potato), it could otherwise feed 600 million to 4 billion people annually, providing 2000 calories per person per day.^13^ The global population is estimated to reach 10 billion by 2050, requiring at least a 60% increase from the current food produced annually.^14^ Reducing the amount of food damaged by fungi is one aspect of the solution to overcome the global food problem and can be achieved by implementing appropriate and novel preventive and management strategies.^13,15^ However, the current chemical fungicide-based approaches are inadequate because plant pathogenic fungi have developed resistance, facilitating global spread.^16^ Besides monoculture farming, another reason for the spread of resistance is climate change, which results in the emergence of pesticide-resistant plant pathogenic fungi in agricultural areas, where they were previously absent. The spread is further accelerated by global trade expansion and intensification, facilitating the introduction of resistant plant pathogenic fungal strains to new agricultural areas.^17^ The problem has been further compounded in Europe by withdrawing and banning effective chemical fungicides from commercial use under the current European Union (EU) legislation on plant protection products.^18^ Moreover, the EU’s action plan (Toward Zero Pollution for Air, Water and Soil), aimed at reducing the environmental burden of chemical pesticides, mandates a 50% reduction in chemical pesticides in Europe by 2030.^19^ Collectively, these factors warrant the development of fungicides with a low environmental impact and minimal risk of resistance development in plant pathogenic fungi.^20^

This review provides a comprehensive picture of the current state and challenges of antifungal therapies and agricultural fungal pest management of the above-mentioned aspects, with a particular focus on insights gained over the past 5 years. Furthermore, it discusses novel developments and solutions to overcome these recently emerging antifungal issues.

## 2 Antifungal Therapy

The severity of the issues associated with currently used antifungal agents for therapeutic purposes is demonstrated by the numerous highly cited review articles published in recent years and over the past decade, highlighting the challenges involved in the development of new antifungal drugs.^21–30^

The treatment of fungal infections in humans currently relies on five antifungal drug classes (polyenes, azoles, allylamines, echinocandins, and others), each possessing different mechanisms of action, with the majority directly or indirectly targeting the cell membrane or cell wall (Table 2).^31^ For the treatment of non-life-threatening superficial skin and toenail fungal infections that do not pose a significant risk to healthy individuals, nearly all classes of antifungal drugs are effective. Terbinafine, an allylamine antifungal, is widely used; however, it is not employed for systemic infections as it primarily accumulates in nails and hair.^32^ However, in severe, life-threatening invasive mycoses, where fungal infections affect the entire body, only azoles, polyenes, echinocandins, and flucytosine remain suitable and are currently widely used.^33^ A significant limitation of flucytosine is the high prevalence of resistance development, necessitating its use exclusively in combination therapy with other antifungal agents rather than as monotherapy.^34^

**Table 2:** Antifungal Drug Classes Commonly Used for Treating Fungal Infections, Including Details of Their Targets, Mechanisms of Action, and Most Frequently Applied Representatives

drug class	representative agents	target	mechanism of action
polyenes	amphotericin B, natamycin, nystatin	cell membrane (direct)	binds to ergosterol, forming pores in the fungal cell membrane, which leads to cell lysis
azoles	imidazoles: clotrimazole, ketoconazole, luliconazole	cell membrane (indirect)	inhibits ergosterol biosynthesis by blocking lanosterol-14α-demethylase, compromising membrane structure and integrity
	triazoles: efinaconazole, fluconazole, isavuconazole, itraconazole, posaconazole, voriconazole		
allylamines	butenafine, naftifine, terbinafine	cell membrane (indirect)	inhibits ergosterol biosynthesis by blocking the enzyme squalene epoxidase, damaging membrane formation and generating toxic squalene byproducts
echinocandins	anidulafungin, caspofungin, micafungin	cell wall (indirect)	inhibits 1,3-β-d-glucan synthesis by targeting 1,3-β-glucan synthase, compromising fungal cell wall formation
others	flucytosine	nucleic acid synthesis (indirect)	interferes with DNA and RNA synthesis via its metabolic products
	griseofulvin	cell division (direct)	binds to tubulin, disrupting microtubule function and inhibiting fungal cell division
	ciclopirox	enzyme cofactors (direct)	chelates polyvalent metal cations, inhibiting the function of enzymes vital for fungal cellular activities
	amorolfine	cell membrane (indirect)	inhibits ergosterol biosynthesis by targeting Δ14-sterol reductase and Δ7–Δ8-cholestenol isomerase, impairing membrane formation

From the 1950s to date, almost exclusively the same early antifungal drug groups (or modified versions) have been used in clinical practice (Figure 1).^27,32^ Furthermore, over the past decade, only four new antifungal agents (2018, superbioavailable itraconazole;[Bibr ref35] 2021, ibrexafungerp;[Bibr ref36] 2022, oteseconazole;^37^ and 2023, rezafungin^38^) have been approved for therapeutic use. However, their mechanisms of action and targets are the same as or similar to those of existing antifungal agents, despite the continuously increasing incidence of infections caused by fungal strains exhibiting resistance to such mechanisms.^39^ Table 3 summarizes these newly introduced antifungal agents and their mechanisms of action. Figure 2 presents the chemical structures of commonly used antifungal drugs.

**Figure 1:**
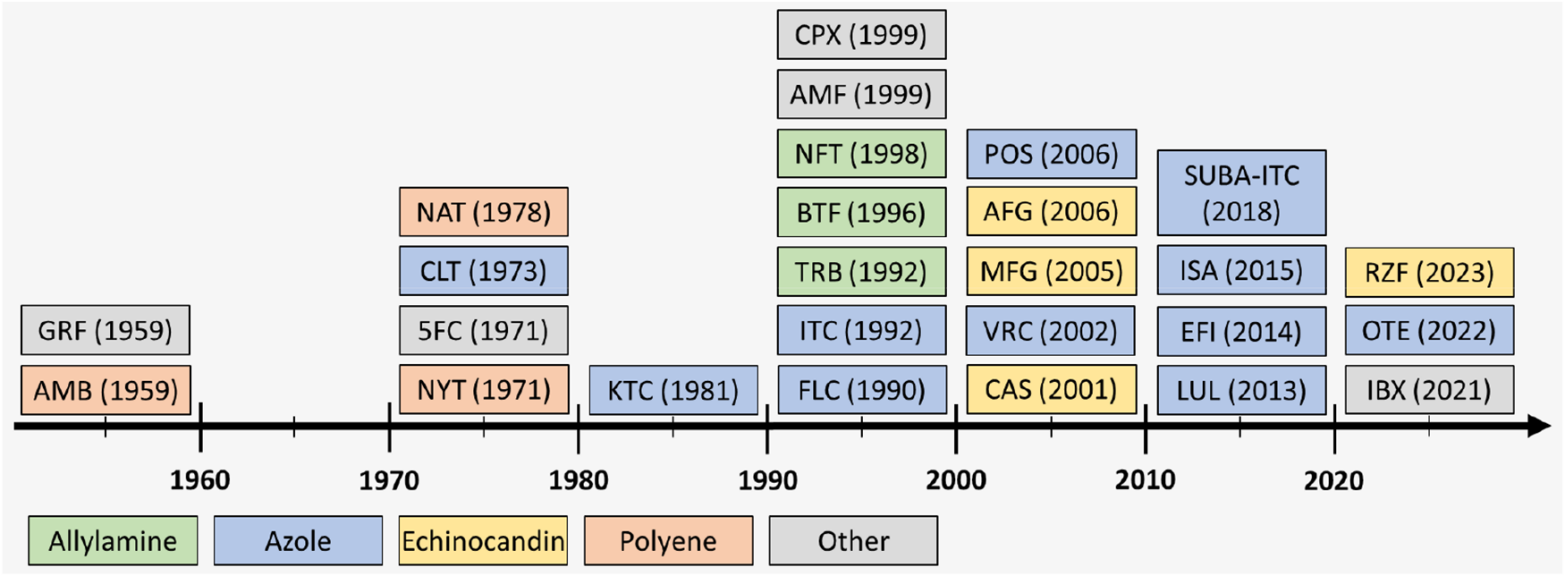
Timeline of antifungal agents approved by the United States Food and Drug Administration (FDA) for general therapeutic use and still in use (up to March 2025). Since the 1950s, antifungal therapies have mainly relied on early drug classes, such as azoles and polyenes or their modified derivatives. Starting from the millennium, echinocandins and ibrexafungerp have emerged as novel antifungal drug classes.^27,32^ However, over the past decade, only four new antifungal agents (superbioavailable itraconazole, ibrexafungerp, oteseconazole, and rezafungin) have been introduced into therapeutic use. This appears counterproductive, because these agents share similar mechanisms of action and targets, such as the fungal cell membrane and cell wall, with existing therapies, despite the increasing prevalence of fungal infections caused by strains resistant to these mechanisms.^39^ 5FC: flucytosine, AMB: amphotericin B, AMF: amorolfine, AFG: anidulafungin, BTF: butenafine, CAS: caspofungin, CLT: clotrimazole, CPX: ciclopirox, EFI: efinaconazole, FLC: fluconazole, GRF: griseofulvin, IBX: ibrexafungerp, ISA: isavuconazole, ITC: itraconazole, KTC: ketoconazole, LUL: luliconazole, MFG: micafungin, NFT: naftifine, NAT: natamycin, NYT: nystatin, OTE: oteseconazole, POS: posaconazole, RZF: rezafungin, SUBA-ITC: superbioavailable itraconazole, TRB: terbinafine, VRC: voriconazole. The figure is compiled using information presented in Houšt’ et al.,^27^ Carmo et al.,^32^ and Zobi and Algul^40^ and updated with the original literature findings.

**Figure 2:**
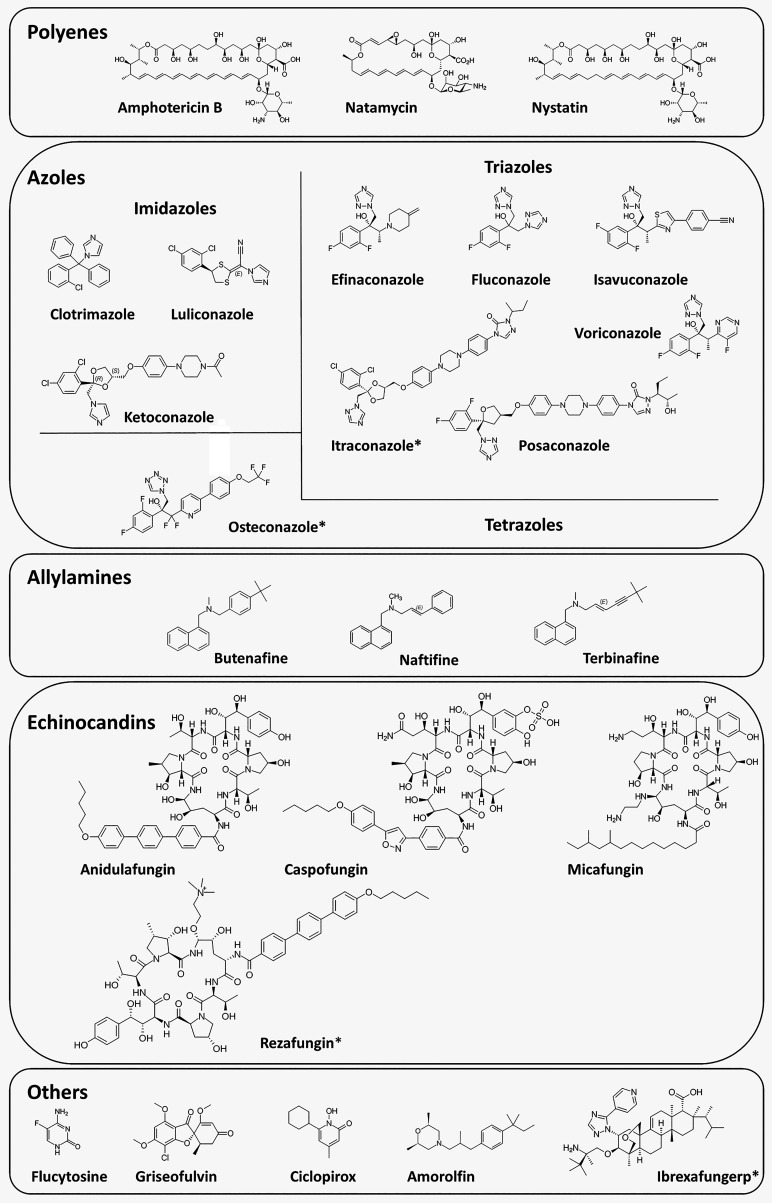
Chemical structures of commonly used antifungal drugs. Asterisks indicate antifungal agents that have been approved for therapeutic use in the past decade.

**Table 3:** Antifungal Agents Approved and Introduced by the United States Food and Drug Administration (FDA) over the Past Decade for Treating Fungal Infections

drug class	representative agents	target	mechanism of action	reference
azoles	triazoles: superbioavailable itraconazole	cell membrane (indirect)	inhibits ergosterol biosynthesis by blocking lanosterol-14α-demethylase, compromising membrane structure and integrity	^35^
	tetrazoles: oteseconazole			^37^
echinocandins	rezafungin	cell wall (indirect)	inhibits 1,3-β-d-glucan synthesis by targeting 1,3-β-glucan synthase, compromising fungal cell wall formation	^38^
others	(triterpenoid): ibrexafungerp			^36^

On one hand, the limited number of safe and effective antifungal agents with new mechanisms of action can be attributed to the lack of interest from pharmaceutical companies. On the other hand, antifungal drug development faces far more challenges than antibacterial drugs, primarily due to the similarities in cell structure, function, and metabolism between fungal and mammalian cells, making it extremely challenging to identify fungal-specific targets.^28,41^ Additionally, developing a new and effective antifungal therapeutic agent must overcome numerous other challenges.

### 2.1 Recent Challenges of Antifungal Therapy

Recent epidemiological studies have demonstrated that the effective treatment of fungal infections can only be achieved by employing fundamentally different strategies from those currently used.^39^ Such new antifungal therapeutic strategies must align with the susceptibility profiles of newly emerging pathogens, resulting from epidemiological changes. Thus, antifungal agents with novel mechanisms of action must be effective against fungal biofilms, have a low risk of resistance development, and possess fungal-specific mechanisms to avoid severe side effects. Furthermore, these agents must exhibit appropriate pharmacokinetic and pharmacodynamic properties to ensure their suitability for treating invasive fungal infections. Other critical requirements are relatively low production costs and accessibility in the regions most affected by fungal infections, often the world’s poorer countries.^41^

#### 2.1.1 Epidemiological Changes

The substantial changes in the epidemiology of fungal infections in recent years are complex and involve numerous underlying causes, such as climate change, the prophylactic and improper use of antifungal agents, and the coronavirus disease 2019 (COVID-19) pandemic. Changes in weather due to climate change in recent years are some of the most striking manifestations of global warming. Fungi are highly adaptable to gradually increasing environmental temperatures, leading to the development of a thermotolerant phenotype. Thermotolerance higher than that of the environment is a virulence factor that renders previously nonpathogenic fungal species infectious. Thus, changing environmental conditions may lead to the spread of fungal species or their vectors to areas where they were previously absent. Extreme weather associated with climate change (i.e., flooding, storms, and hurricanes) significantly promotes the emergence and spread of previously rare fungal infections. Examples of newly emerging and more widely distributed fungal pathogens include *Apophysomyces trapeziformis*, *Blastomyces* spp., *Candidozyma auris* (formerly *Candida auris*), *Coccidioides immitis*, *Cryptococcus deuterogattii*, *Histoplasma* spp., *Paracoccidioides* spp., and *Talaromyces marneffei*.^42^

The increasing use of prophylactic antifungal treatments due to the growing number of immunocompromised and immunosuppressed patients, combined with inadequate or unavailable identification techniques and the agricultural use of antifungal agents that are also used in medicine (such as triazoles), has disrupted the normal human mycoflora and exerted selective pressure, resulting in the emergence of multidrug-resistant strains. Consequently, the epidemiology of common fungal infections, such as candidiasis and aspergillosis, has significantly changed over the past 2 decades, with drug-resistant infections caused by non-albicans *Candida* species and cryptic *Aspergillus* species becoming increasingly common.^5^

The COVID-19 pandemic further amplified the above-mentioned phenomena, primarily due to the immunosuppressive effects of systemic corticosteroid therapy in the treatment of COVID-19. During and after the pandemic, there was a dramatic increase in infections caused by azole-resistant cryptic *Aspergillus* strains, alongside a continued rise in infections caused by non-albicans *Candida* species and a resurgence of *Candida albicans*. Some Asian countries, particularly India, reported a drastic increase in mucormycosis (>47,500 cases), which is caused by members of the order Mucorales, most of which were linked to COVID-19 infection and systemic corticosteroid therapy.^5^

Combating the newly emerging fungal infections resulting from epidemiological changes requires the development of effective antifungal therapies, regular epidemiological surveys, resistance studies, and diagnostic techniques capable of rapidly and effectively identifying newly emerging species.^43^

#### 2.1.2 Resistance, Biofilm Formation, and Tolerance

Resistance to an antifungal agent can be either innate or acquired. Innate resistance is associated with the general physiological characteristics of the fungus, which may render it resistant to the mechanism of action of a specific class of antifungal agents. Thus, even without any acquired resistance mechanism, the sensitivity of individual fungal species (or even different isolates of the same species) to specific antifungal agents may vary. Acquired resistance is caused by epigenetic changes or genomic mutations that alter a property compared with the wild type, making it resistant to the mechanism of action of an antifungal agent.^44^ Common causes of resistance development include prolonged exposure to a suboptimal application of an antifungal agent during clinical therapy^26^ or the agricultural use of antifungal agents (such as triazoles) also used in human medicine.^45^

It has been established that infections caused by fungi resistant to one or more antifungal agents are rapidly increasing.^39,44^ However, recent studies have highlighted a significant rise in the incidence of infections caused by triazole (fluconazole, itraconazole, and voriconazole)-resistant *Aspergillus fumigatus*, multidrug (amphotericin B, echinocandin, fluconazole, and flucytosine)-resistant *Nakaseomyces glabrata* (formerly *C. glabrata*), and *C. auris*, and terbinafine-resistant dermatophytes (*Trichophyton* spp., *Microsporum* spp., and *Epidermophyton* spp.).^8,44^ Acquired resistance to one antifungal agent often leads to resistance to another, a phenomenon known as multidrug resistance. A good recent example is the emergence of azole/echinocandin-resistant *N. glabrata* strains.^46^ Figure 3 summarizes the observed resistance mechanisms.

**Figure 3:**
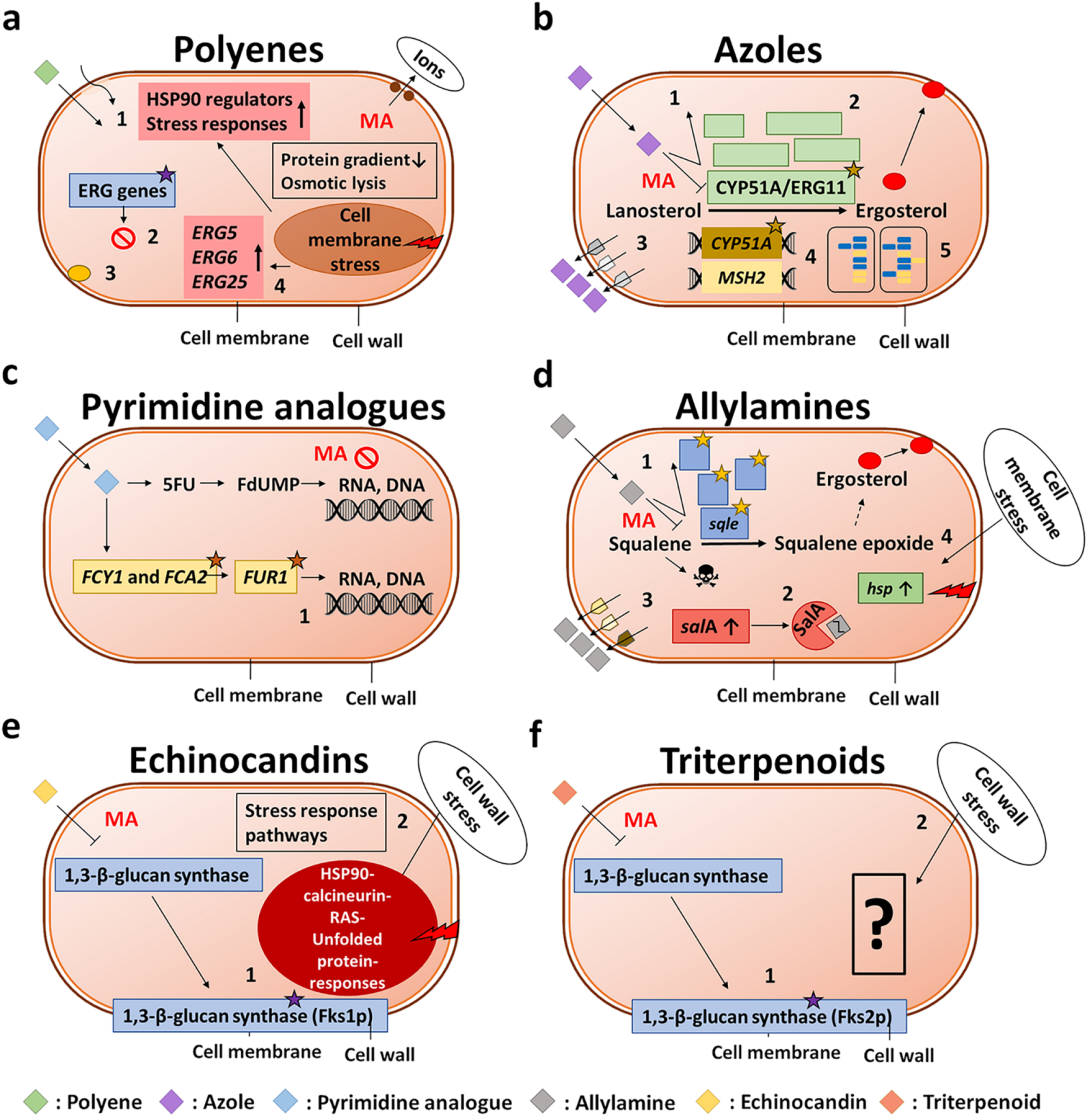
Mechanisms of action of antifungal drug classes and the associated principal forms of acquired resistance. (a) Polyene resistance: (1) altered cell membrane permeability, (2) loss-of-function mutations in ergosterol biosynthesis genes, (3) changes in membrane sterol composition, and (4) induction of genes promoting tolerance and stress response pathways. Mechanism of action (MA): formation of a polyene-ergosterol complex, decreasing the protein gradient and resulting in osmotic lysis.^39^ (b) Azole resistance: (1) point mutations in the gene encoding the target (lanosterol 14α-demethylase), (2) target overproduction, (3) efflux pump overproduction or hyperactivity, (4) hypermutation in the target and housekeeping genes, and (5) heteroresistance and aneuploidy. MA: lanosterol 14α-demethylase inhibition.^39^ (c) Pyrimidine analog resistance: (1) target gene (cytosine deaminase and flucytosine resistance) mutations. MA: RNA and DNA synthesis inhibition.^39^ (d) Allylamine resistance: (1) target gene (squalene epoxidase) mutations, (2) antifungal compound degradation, (3) efflux pump overproduction or hyperactivity, and (4) induction of stress response pathways. MA: squalene epoxidase inhibition.^49^ (e) Echinocandin resistance: (1) point mutations in the gene encoding the target (1,3-β-glucan synthase subunit [*FKS1*]) and (2) cell wall stress response pathway activation. MA: 1,3-β-glucan synthase inhibition.^39^ (f) Triterpenoid resistance: (1) point mutations in the gene encoding the target (1,3-β-glucan synthase subunit [*FKS2*]) and (2) unknown responses to cell wall stress. MA: 1,3-β-glucan synthase inhibition.^50^ 5-FU: 5-fluorouridine, CYP51A/ERG11: lanosterol 14α-demethylase, *ERG*: ergosterol biosynthesis genes, *FCY*: cytosine deaminase gene, *FCA*: flucytosine resistance gene, FdUMP: 5′-fluoro deoxyuridine monophosphate, Fks1p and Fks2p: 3-β-glucan synthase subunits, *FUR1*: uracil phosphoribosyltransferase gene, *hsp*: heat shock protein gene, Hsp90: heat shock protein 90, *MSH2*: azole resistance gene, *sal*A: salicylate 1-monooxygenase gene SalA: salicylate 1-monooxygenase, *sqle*: squalene epoxidase gene. Asterisks indicate mutations. The figure is adapted from Fisher et al.^39^ and updated using information presented in Martinez-Rossi et al.,^49^ and with the original literature findings. Panels (a)–(f) are adapted by permission from Macmillan Publishers Ltd.: NATURE REVIEWS MICROBIOLOGY Fisher, M.C. et al. Tackling the emerging threat of antifungal resistance to human health. *Nat*. *Rev*. *Microbiol*. **2022**, 20(9), 557–571, copyright 2022.

Polyenes alter the cell membrane permeability by forming complexes with ergosterol. Typically, polyene resistance involves loss-of-function mutations in genes involved in the ergosterol biosynthesis pathway, particularly in *Aspergillus* and *Candida* species. In *C. albicans*, the loss of sterol C-5-desaturase (*ERG3*) and the upregulation of sterol C-22-desaturase (*ERG5*), Δ(24)-sterol C-methyltransferase (*ERG6*), and sterol C-4-methyl oxidase (*ERG25*) commonly contribute to polyene resistance (Figure 3a).^39^ The resistance to amphotericin B is rare compared to azoles and echinocandins due to the high fitness cost and the resulting impairment of fungal growth, and reduced survival in host environments.^47,48^

Azoles inhibit ergosterol biosynthesis by targeting lanosterol 14α-demethylase (*ERG11*), disrupting cell membrane formation. Azole resistance is typically caused by efflux pump overexpression in *Candida* species or lanosterol 14α-demethylase mutations and promoter insertions affecting the sterol biosynthesis pathway in *Aspergillus* species. In other species such as *Cryptococcus neoformans*, lanosterol 14α-demethylase overproduction, efflux pump overexpression, and hypermutations are responsible for azole resistance. Additionally, heteroresistance and aneuploidy further facilitate azole resistance (Figure 3b).^39^

Byproducts of pyrimidine analogue metabolism inhibit DNA and RNA synthesis. Resistance to these agents (e.g., flucytosine) mainly arises from point mutations in the target gene cytosine deaminase (*FCY1*), particularly in *Candida* species, and hypermutation is a frequent resistance mechanism in *Cryptococcus* species (Figure 3c).^39^

Allylamines inhibit squalene epoxidase, a key enzyme in the ergosterol biosynthesis pathway, disrupting cell membrane formation. The inhibition of squalene epoxidase leads to the accumulation of toxic squalene byproducts that compromise organelle membrane integrity and ultimately impair fungal viability, which is the primary antifungal mechanism of allylamines. Resistance mechanisms (especially in *Trichophyton* spp.) include mutations in the squalene epoxidase gene, overproduction of salicylate 1-monooxygenase capable of degrading allylamine, efflux pump overexpression, and induction of membrane and stress response pathways (Figure 3d).^49^

Echinocandins and triterpenoids inhibit 1,3-β-glucan synthase, preventing the synthesis of 1,3-β-d-glucan, a cell wall component.^39,50^ Echinocandin resistance typically results from mutations in 1,3-β-glucan synthase (*FKS1*), particularly in *Candida* and *Fusarium* species (Figure 3e).^39^ Triterpenoid resistance is also associated with mutations in 1,3-β-glucan synthase, specifically impacting *FKS2* in *N. glabrata* (Figure 3f).^50^ Stress response pathways, such as those involving heat shock protein 90 (Hsp90), Ca^2+^/calcineurin signaling, Ras GTPase-linked mechanisms, and the unfolded protein response, have been implicated in echinocandin resistance (Figure 3e).^39^ However, the role of these pathways in triterpenoid resistance mechanisms has yet to be fully elucidated (Figure 3f).^50^

A notable factor contributing to antifungal resistance is the biofilm-forming properties of fungi. Most human pathogenic fungi form biofilms within the human body or on medical devices and instruments. These comprise a mass of fungal cells adhered to a surface and embedded in an extracellular matrix of polymer compounds secreted by the fungi. Biofilm formation begins with fungal cells or spores adhering to a living or inorganic surface, followed by filamentous growth, interconnection, and secretion of extracellular matrix materials, forming a protective barrier. This matrix masks the fungal cells, providing extensive protection against the host’s immune system and antifungal agents.^51^ This is further enhanced by increased efflux pump activity within the biofilm, changes in antifungal targets within fungal cells, and the effects of proteins associated with filamentation.^52^ In biofilm form, fungi sensitive in the planktonic (yeast-like) state may become resistant to antifungal agents.^53^

These observations indicate the urgent need for novel antifungal agents with minimal risk of resistance development (or at least very slow resistance development) and effectiveness against fungal biofilms.

Recent studies have shown that in addition to genetic resistance, phenotypic resistance (antifungal drug tolerance) also affects the therapeutic efficacy of antifungal drugs. Antifungal drug tolerance, distinct from resistance, is characterized by the slow proliferation of subpopulations that efficiently overcome antifungal stress. In *C. albicans*, tolerance exhibits an inverse correlation with intracellular drug accumulation and is mitigated by adjuvant compounds administered alongside fluconazole, thereby enhancing therapeutic efficacy in highly tolerant isolates.^54^ In *C. neoformans*, brain glucose induces tolerance to amphotericin B via the glucose repression activator Mig1, ultimately reducing treatment effectiveness in cryptococcal meningitis. Mig1-driven tolerance mechanisms involve suppression of ergosterol synthesis and augmented production of inositolphosphorylceramide, which competes with amphotericin B for ergosterol binding. Co-administration of amphotericin B with aureobasidin A, a fungal-specific inositolphosphorylceramide synthase inhibitor, significantly enhances antifungal efficacy.^55^ Additionally, the *C. neoformans* develops fungicide-tolerant persisters in pulmonary infections enriched in cells with high stationary-phase molecule production. The antioxidant ergothioneine plays a crucial role in amphotericin B persistence, indicating a conserved tolerance mechanism across diverse fungal species. Sertraline demonstrates selective antifungal activity against amphotericin B-tolerant persisters, suggesting a promising therapeutic avenue for cryptococcosis.^56^ Understanding fungal tolerance mechanisms can improve antifungal therapy strategies by identifying new drug targets and optimizing combination treatments.

#### 2.1.3 Safe and Effective Applicability

The effective and safe application of an antifungal agent requires a thorough understanding of its pharmacokinetic and pharmacodynamic properties, which may vary among different classes of antifungal drugs. Taking these into consideration, along with the susceptibility of the pathogenic fungus to antifungal agents, certain antifungal drugs are suitable for treating different types and localizations of fungal infections.^32^ Severe side effects (mainly kidney and liver damage) are prone to developing during the treatment of invasive fungal infections, particularly prolonged intravenous administration of antifungal agents such as polyenes, triazoles, and flucytosine. Less severe side effects (e.g., arrhythmias and disorders of the central nervous system, cardiovascular system, and respiratory system) must also be considered for all antifungal drugs. Echinocandins are currently considered the safest class of antifungal drugs.^57^ However, because they have significantly poorer pharmacokinetic and pharmacodynamic properties when administered orally, they are only available for intravenous use.^32^ Table 4 summarizes the pharmacokinetic and pharmacodynamic properties of the antifungal agents used to treat life-threatening invasive fungal infections.^32,58–60^

**Table 4:** Pharmacokinetic and Pharmacodynamic Characteristics of the Antifungal Agents Commonly Used for Treating Invasive Fungal Infections^a^

drug class	application	pharmacokinetics	pharmacodynamics	central nervous system penetration	key efficacy	serious side effects
polyenes (amphotericin B)	topical	bioavailability (oral administration): low	concentration-dependent antifungal effect: yes	cerebrospinal fluid: low	*Aspergillus* spp., *Blastomyces dermatitidis*, *Candida* spp., *C. immitis*, *C. neoformans*, *Histoplasma capsulatum*, *Mucor* spp., *Rhodotorula* spp., *Sporothrix schenckii*	nephrotoxicity
	oral	protein binding: high	prolonged post-antifungal effect: yes	brain tissue: low		
	intraperitoneal	metabolism (cytochrome P450): none	efficacy: concentration-dependent			
	intravenous	excretion (unmetabolized): renal-low, hepatic-high				
		distribution: plasma, extracellular fluids, outside the bloodstream				
		clearance: moderate				
		half-life: medium to long				
		time to maximum drug concentration: medium				
triazoles (fluconazole, itraconazole, posaconazole, voriconazole, isavuconazole)	topical	bioavailability (oral administration): high	concentration-dependent antifungal effect: no	cerebrospinal fluid (%): low to high	*Aspergillus* spp., *B. dermatitidis*, *Candida* spp., *C. immitis*, *C. neoformans*, *H. capsulatum*, *Paracoccidioides brasiliensis*, *Phaeohyphomycetes* spp., *S. schenckii*	hepatotoxicity
	oral	protein binding: low to high	prolonged post-antifungal effect: yes	brain tissue (%): high		
	intraperitoneal	metabolism (cytochrome P450): yes or glucuronidation	efficacy: exposition and minimum inhibitory concentration-dependent			
	intravenous	excretion (unmetabolized): renal-medium to high, hepatic-low to high				
		distribution: outside the bloodstream				
		clearance: high				
		half-life: medium to long				
		time to maximum drug concentration: short to medium				
echinocandins (anidulafungin, caspofungin, micafungin)	intravenous	bioavailability (oral administration): orally not administered	concentration-dependent antifungal effect: yes	cerebrospinal fluid: low	*Candida* spp.	not known
		protein binding: high	prolonged post-antifungal effect: yes	brain tissue: low to medium		
		metabolism (cytochrome P450): yes, spontaneous degradation, independent	efficacy: exposition and (minimum inhibitory) concentration-dependent			
		excretion (unmetabolized): renal-low, hepatic-low				
		distribution: plasma, extracellular fluids, outside the bloodstream				
		clearance: high				
		half-life: medium to long				
		time to maximum drug concentration: short to medium				
flucytosine	oral	bioavailability (oral administration): high	concentration-dependent antifungal effect: no	cerebrospinal fluid: high	*Candida* spp., *C. neoformans*	nephrotoxicity, hepatotoxicity, bone marrow damage
	intravenous	protein binding: low	prolonged post-antifungal effect: no	brain tissue: high		
		metabolism (cytochrome P450): minimal	efficacy: time-dependent			
		excretion (unmetabolized): renal-high				
		distribution: plasma, extracellular fluids				
		clearance: moderate				
		half-life: short				
		time to maximum drug concentration: medium				

a Compiled based on tables from Mazzei and Novelli,^58^ Lepak and Andes,^59^ Carmo et al.,^32^ and Ashley.^60^

The effective treatment of central nervous system fungal infections is exceptionally challenging, mainly due to the limited penetration of currently available antifungal agents across the blood–brain barrier, which can be significantly improved using liposomal formulations (e.g., amphotericin B) (Table 4). However, therapy remains unsuccessful in most cases, even when the theoretically effective dose of the antifungal agent is predicted to enter the central nervous system because traditional pharmacokinetic principles are not universally applicable to the central nervous system. Furthermore, altered physiology in immunosuppressed patients and the use of other medications influence the pharmacokinetics and pharmacodynamics of antifungal drugs.^60,61^ Consequently, central nervous system fungal infections are associated with high mortality rates regardless of the pathogen, reaching 90–100% in immunosuppressed patients.^62^

One of the causes of the development of severe side effects is the similarity in cell structure, function, and metabolism between fungi and human hosts. Thus, the development of exclusively fungus-specific antifungal agents is challenging. However, this problem could be solved by identifying fungus-specific molecular targets. Some fungi enter host cells, where they are less exposed to the effects of antifungal agents and the immune system. Therefore, effective antifungal agents or therapeutic approaches must have good cell penetration properties.^41^ These observations emphasize the urgent need to develop small-molecule antifungal compounds capable of penetrating the blood–brain barrier, ensuring both safe application and predictable therapeutic efficacy.^61^

#### 2.1.4 Cost and Availability

Introducing a compound with proven antifungal effects for clinical therapeutic use is expensive and time-consuming. In 2015, it was estimated that this process would take approximately 10 years and cost around $300 million in the United States, with an additional $400 million in marketing expenses for commercial launch.^63^ Furthermore, a 2020 study estimated that developing an entirely new drug (including target identification and approval) would take 12–20 years, costing approximately $1.8 billion.^64^ Considering the rapid spread of resistance to currently used antifungal agents, it is conceivable that these costs may never be recovered before the drug becomes unsuitable for therapeutic use. Coupled with the current estimate of only a 5% chance of newly developed antifungal molecules passing the clinical testing phases, the interest of pharmaceutical companies in developing and marketing new antifungal agents is limited.^41,64^

The availability, cost, and therapy expenses of different antifungal agents vary across countries.^65^ This disparity significantly affects the ability of middle- and, most notably, low-income countries, which are the most heavily affected regions in terms of life-threatening fungal infections, to effectively combat fungal infections.^66^ A prime example is the prohibitive cost and limited availability of liposomal amphotericin B and flucytosine therapies in these countries.^67^ Furthermore, the lack of proper diagnostic knowledge and tools, as well as mitigating surveys, contribute to the exceptionally high number of fatal mycoses in these regions.^66,68^ To alleviate this problem, policies must be implemented to ensure that regardless of cost, the most effective antifungal agents and diagnostic techniques are globally accessible. Moreover, strict guidelines for effectively using antifungal agents must be established, requiring a well-trained workforce of specialists.^66^

### 2.2 New Trends in Antifungal Therapy

A potential solution to the antifungal problems is the combined use of antifungal drugs using the simultaneous or sequential application of two antifungal agents with distinct mechanisms of action and targets. This approach reduces the risks of resistance development and side effects, while synergistically enhancing each other’s efficacy and broadening the antifungal spectrum.^69^ Over the past 3 decades, numerous drugs used to treat different diseases have been shown to possess secondary antifungal effects beyond their original indication, making them potentially useful as either independent or adjuvant treatments. Repurposing such drugs for antifungal application is cheaper and quicker than developing an entirely new agent.^70^ Using drug databases to identify targets suitable for fungus-specific antifungal therapy enables the design of new drug molecules or drug repurposing, laying the groundwork for safe, side-effect-free antifungal therapies in the future.^71^ Several promising candidate molecules possessing novel antifungal mechanisms of action have already reached various stages of clinical testing. However, they have yet to be introduced for therapeutic use.^32^

#### 2.2.1 Antifungal Drug Combinations

The interaction between two combined antifungal agents is either synergistic (mutually enhancing each other’s efficacy), additive (equaling the sum of their individual efficacies), or antagonistic (mutually reducing each other’s efficacy). Synergy is favorable for achieving a more effective antifungal treatment than monotherapy, but antagonism should be avoided. Notably, the efficacy of an antifungal agent can be enhanced not only by another antifungal drug but also by a drug not known for its antifungal properties. Such an example is ibuprofen, a nonsteroidal anti-inflammatory drug, which, despite lacking inherent antifungal activity, synergistically increases the efficacy of fluconazole against *Candida* infections by efflux pump inhibition. A synergistic antifungal drug combination involves one of the following underlying mechanisms: (1) one agent increases the biological availability of the other, (2) the agents target different components of the same biological pathway or different but interrelated pathways, or (3) one agent suppresses the stress response induced by the other. An example of the first mechanism is the interaction between amphotericin B and flucytosine, where amphotericin B enhances membrane permeability, facilitating the intracellular uptake of flucytosine. An example of the second mechanism is the interaction between terbinafine and azoles, where both target the ergosterol biosynthesis pathway but at different points (Figure 3b,d). An example of the third mechanism is the combined application of Hsp90 inhibitors, which block the stress response to azoles. Because these synergistic antifungal combinations, often only observed *in vitro*, have the potential for therapeutic success, many new methods have recently been developed to identify synergistic antifungal drug combinations.^72^ In clinical practice, the most frequently used drug combinations for therapeutic purposes include amphotericin B + flucytosine/azoles or echinocandin + azoles for invasive candidiasis, azoles/amphotericin B + echinocandin or voriconazole + anidulafungin for invasive aspergillosis, and amphotericin B + posaconazole/caspofungin for mucormycosis.^73^

#### 2.2.2 Drug Repurposing

Drug repurposing involves identifying new therapeutic applications for commercially available drugs beyond their original use. Such an approach offers numerous advantages and may be the basis for a novel antifungal strategy. Including commercial market authorization, this process only takes 3–12 years and costs approximately $50 million, a fraction of the outlay required to develop an entirely new drug.^54^ Various *in vitro* and *in silico* techniques have identified numerous candidate antifungal drug molecules from drug molecule databases, primarily against *Candida* species.^64,70,74^ Potential candidates for repurposed antifungal agents include various antibiotics, immunosuppressive drugs, cholesterol-lowering statins, antiarrhythmic drugs, antipsychotics, antidepressants, and nonsteroidal anti-inflammatory drugs.^70^ However, repurposed antifungal agents have several drawbacks: (1) *in vitro* antifungal activity does not guarantee *in vivo* efficacy, (2) therapeutically effective antifungal doses may cause severe side effects, (3) low concentrations may lead to resistance development, and (4) the exact mechanism of their antifungal action is not always fully understood, hindering therapeutic applications.^64,67,74^ Nevertheless, even in unsuccessful applications, these drugs may serve as templates for developing new antifungal agents.^67^

#### 2.2.3 Identification of Novel Fungal-Specific Targets

Developing a fundamentally new and safe antifungal agent begins by identifying fungal-specific targets that differ from those targeted by commercially available antifungals. These novel targets enable the selective destruction of pathogenic fungi, potentially reducing the risk of side effects in humans.^21^ Such novel fungus-specific targets may be components of pathways already affected by commercially available antifungal agents (e.g., ergosterol biosynthesis and cell wall biosynthesis) that have yet to be targeted or entirely new pathways. As mentioned earlier, newly identified targets may also be used for drug repurposing. A major advantage of developing new agents targeting components of pathways already addressed by commercial antifungal agents is that drugs with similar mechanisms of action have already shown effectiveness in clinical settings, thereby increasing the likelihood of these new agents successfully passing clinical testing phases.^75^ Newly targeted pathways primarily include those involved in resistance development (e.g., efflux pumps, lanosterol 14α-demethylase expression, and Hsp90), virulence (e.g., biofilm formation and morphological changes), respiration (mitochondria), and metabolism (e.g., various enzymes of gluconeogenesis).^40,71^ Pathways to consider from these aspects include Hsp90-calcineurin-protein kinase C-linked pathways (e.g., Hsp90-associated proteins and FK506-binding protein 12), Ras GTPase signaling pathways (e.g., diaminobutyrate-2-oxoglutarate, aminotransferase, and farnesyltransferase), other signaling pathways (MAP kinase, phosphoinositide-dependent kinase, sphingosine 1-phosphate), the glyoxylate cycle (e.g., isocitrate lyase and malate synthase), trivalent cation-linked pathways (e.g., polyketide synthase and siderophore synthase), the trehalose biosynthesis pathway (e.g., trehalose-6-phosphate synthase), the lipid biosynthesis pathway (e.g., ceramide synthase and serine palmitoyl transferase), aspartate pathway and amino acids biosynthesis-related enzymes (e.g., acetohydroxyacid synthase, aspartate transaminase), pyrimidine pathway (e.g., dihydroorotate dehydrogenase), and the respiratory chain (e.g., mitochondrial cytochrome bc1-reductase and cytochrome P450).^40,76,77^ Besides these, numerous other potential targets are under consideration, including transcription factors (e.g., sterol uptake control protein 2), ceramide synthase, acetyltransferases (e.g., inositol acyltransferase), deacetylases (e.g., histone deacetylases playing a role in heterochromatin remodelation), and siderophores.^40,75,77^

#### 2.2.4 Development of Entirely New Antifungal Drugs

The first step in developing an entirely new antifungal drug involves screening a broad molecular library for *in vitro* antifungal activity followed by identifying its target fungal component. Then, the molecule’s structure may be optimized to enhance its efficacy, and it is tested in mammalian infection models. If proven effective in these models, the drug undergoes phases of clinical testing, followed by commercialization if it passes the trials.^41^ Table 5 provides an overview of the antifungal agents developed to target components of old and newly targeted biological pathways, which were still undergoing clinical testing at the time of writing of this review (March 2025). Thus, it is likely that many of these agents have now passed the final clinical test phase and been approved and commercially launched, especially those that are in the third phase of clinical trials focusing on the effectiveness against existing treatments and monitoring the adverse reactions. Entirely new antifungal agents in the third phase of clinical trials include fosmanogepix, olorofim, chelated amphotericin B, albaconazole, iodiconazole, and opelconazole.^40^ Fosmanogepix inhibits glycosylphosphatidylinositol-linked cell wall transfer protein 1 (Gwt1), preventing essential changes required for cell survival. This mechanism differs entirely from the previous antifungal drugs used in clinical settings.^78^ Olorofim, a dihydroorotate dehydrogenase inhibitor, disrupts pyrimidine biosynthesis but is ineffective against *Candida* species. This mechanism is also completely different from those of currently used antifungal agents.^79^ Chelated amphotericin B removes ergosterol from the cell membrane by binding to it and forming aggregates. This formulation differs from existing amphotericin B preparations because it achieves high bioavailability, even with oral administration.^80^ Albaconazole, iodiconazole, and opelconazole are new triazoles with a broader spectrum of antifungal activity and stronger binding to lanosterol 14α-demethylase than existing triazoles.^40,81^ Figure 4 presents the chemical structures of the antifungal drugs under development.

**Figure 4:**
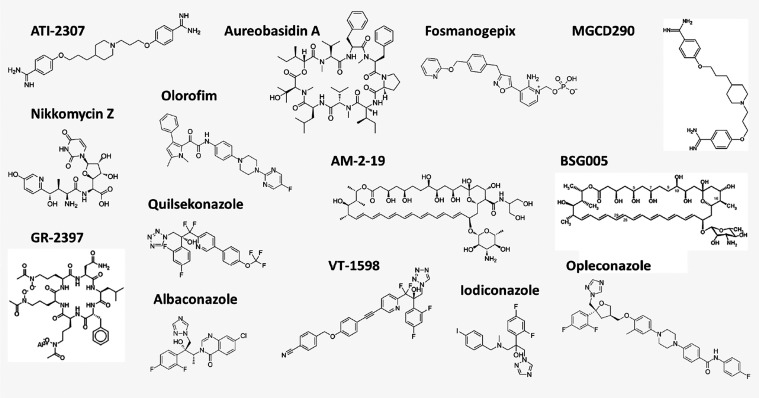
Chemical structures of antifungal drugs under development.

**Table 5:** Antifungal Drugs under Development^a^

drug class	drug	mechanism of action	application	spectrum	advantages	clinical trial phase
arylamidine	ATI-2307	inhibition of mitochondrial complex III and IV enzymatic activity, decreasing ATP production	intravenous	*Aspergillus* spp., *Candida* spp., *Cryptococcus* spp., *F. solani*, *Malassezia furfur*	effective against all FPPL pathogens, better bioavailability	phase I
depsipeptide	aureobasidin A	inhibition of inositol-phosphoceramide synthase	oral, intravenous	*Aspergillus* spp., *Candida* spp.	broad antifungal spectrum	preclinical
GPI inhibitor	fosmanogepix (APX001)	inhibition of GPI-anchored cell wall transfer protein 1 (Gwt1)	oral, intravenous	*Aspergillus* spp., *Candida* spp., *Coccidioides* spp., *Fusarium* spp., *Rhizopus arrhizus*, *Scedosporium* spp.	effective against antifungal-resistant *C. albicans* and *C. auris* strains	phase III
histone deacetylase inhibitor	MGCD290	reduction of stress response reactions through inhibition of histone deacetylase and Hsp90	oral	*Aspergillus* spp., *Candida* spp., *Fusarium* spp., *Mucor* spp., *Rhizopus* spp., *Rhodotorula* spp., *Trichosporon* spp., *Scedosporium apiospermum*	synergistic interaction with echinocandins and azoles	phase II
nucleoside peptide	nikkomycin Z	inhibition of type 1 chitin synthase	oral	*B. dermatitidis*, *Candida* spp., *Coccidioides* spp., *H. capsulatum*, *Sporothrix globosa*	synergistic interaction with echinocandins and itraconazole against *C. albicans*, *C. parapsilosis*, *C. neoformans*	phase I/II
orotomide	olorofim (F901318)	inhibition of pyrimidine biosynthesis via dihydroorotate dehydrogenase inhibition	oral, intravenous	*Aspergillus* spp., *B. dermatitidis*, *Coccidioides* spp., *Fusarium* spp., *H. capsulatum*, *L. prolificans*, *Microsporum gypseum*, *Penicillium* spp., *Pseudallescheria boydii*, *S. apiospermum*, *S. schenckii*, *Trichophyton* spp.	effective against antifungal-resistant fungi	phase III
polyene (analog)	AM-2-19	removal of ergosterol from the cell membrane via aggregation	nd	*Aspergillus* spp., *Candida* spp., *Cryptococcus* spp., *Coccidioides* spp., *H. capsulatum*	effective against amphotericin B-resistant *Aspergillus* strains. Does not bind to cholesterol	*in vivo* experiments
	BSG005		intravenous	*Aspergillus* spp., *Candida* spp., *Cryptococcus* spp., *Mucor* spp., *Pneumocystis* spp.	fungicidal activity against antifungal-resistant *Aspergillus* and *Candida* isolates. Less nephrotoxic than amphotericin B	phase I
	chelated amphotericin B (CAmB)		oral	*Aspergillus* spp., *Blastomyces* spp., *Candida* spp., *Coccidioides* spp., *Fusarium* spp., *Histoplasma* spp., *Paracoccidioides* spp., *Scedosporium* spp.	improved bioavailability	phase III
siderophore (analogue)	GR-2397 (ASP2397, VL-2397)	unknown, uptake through Sit1 transporter	intravenous	*Aspergillus* spp., *Candida* spp., *F. solani*, *Trichosporon asahii*	effective against azole-resistant *Aspergillus* strains	phase II
tetrazole	quilsekonazole (VT-1129)	inhibition of ergosterol biosynthesis via lanosterol 14α-demethylase inhibition	oral	*Candida* spp., *Coccidioides* spp.	specific for fungal lanosterol 14α-demethylase	preclinical
	VT-1598			*Aspergillus* spp., *B. dermatitidis*, *Candida* spp. *Coccidioides* spp., *C. neoformans*, *H. capsulatum*, *R. arrhizus*		phase I
triazole	albaconazole		oral	*Aspergillus* spp., *Candida* spp., *Cryptococcus* spp., *Nannizzia gypsea*	broad antifungal spectrum. Good bioavailability	phase III
	iodiconazole		topical	*Aspergillus* spp., dermatophyte species	broad antifungal spectrum. High binding affinity to lanosterol 14α-demethylase	phase III
	opelconazole		inhalation	*Aspergillus* spp.		

a ATP: adenosine triphosphate, FPPL: fungal priority pathogens list,^10^ GPI: glycosylphosphatidylinositol, Hsp90: heat shock protein 90, nd: no data available. Compiled based on tables from Pfaller et al.,^77^ Bouz and Doležal,^35^ Puumala et al.,^41^ and Zobi and Algul.^40^

#### 2.2.5 Other Therapeutic Options (Biopharmaceutical Products and Nanoparticles)

Other antifungal therapeutic options include those based on biopharmacological products (e.g., monoclonal antibodies, cytokines, vaccines, or antifungal peptides) or nanoparticles.^62^ Because monoclonal antibodies are highly target-specific, their use as antifungal therapeutics offers a significant advantage, making them a potentially safe option with minimal side effects. Examples of effective monoclonal antibodies include efungumab and 18B7. However, despite several other monoclonal antibodies showing both *in vitro* and *in vivo* efficacy, no such products are currently under development for clinical use, which may be due to the high cost of production technology.^75^

Cytokines (e.g., recombinant macrophage colony-stimulating factor, interferon-γ) or other proteins may be used in immunotherapeutic antifungal strategies. Cytokines stimulate the host’s immune system, enhancing defense against fungal infections. However, more extensive and complex clinical trials are necessary to understand their mechanisms of action and ensure safe application for treating fungal infections. Recombinant macrophage colony-stimulating factor is a promising candidate that has long been used for the clinical treatment of neutropenia.^75^

Vaccination is another encouraging option to prevent fungal infections and spread, particularly in regions where pathogenic fungi pose a significant issue and hospital treatment is either prohibitively expensive or unavailable (typically low-income countries). Because it is challenging to enhance immune defense in immunosuppressed patients through vaccination, vaccines are primarily envisioned as prophylactic agents for use in immunocompetent populations.^82^ No commercially available antifungal vaccines exist, but two are under development and have shown promising results in clinical trials. The NDV-3A vaccine comprises the N-terminal domain of a protein with agglutinin-like sequences (Als3) combined with an aluminum hydroxide adjuvant. Als3 is an immunodominant cell wall protein of *C. albicans* that functions as a virulence factor. NDV-3A has demonstrated effectiveness in phase II clinical trials for treating infections caused by various *Candida* species. Its mechanism of action is based on reducing pathogenic virulence, while enhancing the production and activation of phagocyte effectors. The recombinant NXT-2 vaccine is based on a modified Kex1 protein (serine-type carboxypeptidase) designed to achieve protection against a broad antifungal spectrum by stimulating the production of anti-NXT-2 antibodies, which bind to fungal cells and promote phagocytosis. Additional advantages include inhibiting biofilm formation and cost-effective production, and tests indicate that it may be effective against *A. fumigatus*, *C. albicans*, *C. neoformans*, and *Pneumocystis jirovecii* infections. It is currently undergoing phase I clinical trials.^41^

Antifungal peptides offer several advantages over traditional antifungal agents. They are naturally derived, making them potential biopharmacological products. They act across a broad spectrum, enabling their effectiveness against various fungal infections. Furthermore, their selective mode of action targets fungi, while minimizing the risk of side effects. Moreover, the risk of resistance development is minimal because they typically have a rapid mode of action and are fungicidal, attacking multiple fungal-specific targets using diverse mechanisms.^83–85^ Generally, the mechanism of action is dependent on the peptide concentration. At relatively high concentrations, antifungal peptides directly disrupt the cell membrane, causing rapid cell death. They exert various effects at lower concentrations, including directly or indirectly inhibiting cell wall synthesis, binding to nucleic acids to cause degradation, suppressing transcription, disrupting organelle function (e.g., mitochondria and vacuoles), inducing reactive oxygen species accumulation, triggering programmed cell death and autophagy, disturbing ion homeostasis, altering fungal metabolism, activating signal transduction pathways, and interfering with the cell cycle.^86,87^ Despite their many advantages, antifungal peptides also have several drawbacks that significantly affect their practical applicability, including high instability within the host organism (mainly due to enzymatic degradation), host cell lysis when applied at high concentrations, unresolved drug delivery and targeting, low yield, and high production cost. Due to their unfavorable pharmacokinetic and pharmacodynamic properties, the medical application of antifungal peptides is currently envisioned in the form of topical agents for the treatment of various types of candidiasis, either as a stand-alone therapy or in combination with traditional antifungal agents.^87–89^ Several such peptides have reached various stages of clinical trials, but only a few have been commercialized as therapeutic agents. For instance, the peptide P113 demonstrated effectiveness against oral, vaginal, and ocular candidiasis, and NP213 showed promise for treating onychomycosis, although it failed placebo-controlled tests.^83^ Regarding systemic applications for treating invasive mycoses, hLF(1–11) and CZEN-002 are promising candidates. hLF(1–11) directly destroys pathogens, while CZEN-002 enhances the host’s immune defense. Despite promising clinical trials, neither has been commercialized to date.^83^ The previously mentioned and development-stage agents, such as nikkomycin Z, aureobasidin A, and VL-2397, are broadly effective in treating fungal infections of diverse origins and types.^84^ Additional promising candidates for treating *Candida* infections include cathelicidins (e.g., LL-5, LL-37, omiganan, and iseganan), histatins (e.g., histatin 5), mucins (e.g., PAC113), and other peptides (e.g., lactoferrin, WLBU2, and XF-73), many of which have already reached various stages of clinical trials.^28,90^ Two lactoferrin-derived peptides (i.e., Lf(1–11) and bLfcin) are notable as potential adjuvants because they exhibit synergistic effects against *Candida* species when combined with azoles or amphotericin B70. Moreover, many antifungal peptides have shown effectiveness in preventing biofilm formation and destroying already existing biofilms. This is mainly achieved by inhibiting surface adhesion and negatively regulating genes involved in the complex processes of biofilm formation, such as those regulating the transition from planktonic cells to pseudohyphae to hyphae. Such peptides include certain defensins, cathelicidins, histatins, and *de novo*-designed peptides.^91^

The application of nanotechnology in treating fungal infections has emerged in recent years. This involves binding antifungal agents to nanoparticles or packaging in nanocapsules to improve their pharmacokinetic and pharmacodynamic properties. Examples include lipid nanoparticles, such as amphotericin B in liposomal bilayers (AmBisome), or nanopolymers. Metallic nanoparticles (e.g., silver, gold, and magnetite) have also demonstrated effective antifungal activity, although their clinical application remains unelucidated.^31^

#### 2.2.6 Artificial Intelligence (AI) in Antifungal Drug Development

These days, artificial intelligence (AI) is revolutionizing antifungal drug development and therapies, reducing time and costs while enhancing predictive models for efficacy and safety. Additionally, AI facilitates biomarker identification and personalized treatment strategies, leading to more effective and tailored antifungal therapies. Machine learning models surpass traditional screening methods by processing genomic, proteomic, and metabolomic data to identify promising drug candidates. AI-driven predictive modeling assesses drug interactions, toxicity profiles, and pharmacokinetic properties, optimizing development processes.^35,92^ AI also aids in identifying resistance patterns in fungal pathogens, enabling the design of targeted therapies for multidrug-resistant strains, accelerating the discovery of novel treatments.^41^ AI plays a key role in drug repurposing for antifungal applications by analyzing molecular structures and predicting efficacy. Leveraging clinical data sets and natural language processing, AI discovers new therapeutic indications for existing antifungal agents.^35,92^ Advanced techniques, such as reinforcement learning and generative adversarial networks, further refine drug effectiveness and enhance therapeutic potential, improving accessibility, efficacy, and affordability. AI-driven screening methods identify agents with improved efficacy and lower toxicity while optimizing formulations and refining safety models. Machine learning algorithms contribute to streamlining clinical trials, improving therapeutic strategies, and increasing treatment success rates. Personalized antifungal treatment is enhanced by AI through analyzing individual patient data, optimizing therapeutic efficacy, and minimizing adverse effects. By integrating genomic information, microbiome composition, and treatment history, AI-driven models assist clinicians in selecting the most effective therapies and improving treatment precision, patient adherence, and clinical outcomes. AI further enables real-time monitoring of patient responses, allowing timely therapeutic adjustments.^41,92^ Despite its transformative potential, AI integration in antifungal drug development faces challenges, including limited high-quality data sets, regulatory hurdles, and ethical concerns regarding data privacy and accessibility. Addressing these challenges through interdisciplinary collaboration, standardized data collection, and clear validation guidelines will improve the reliability of AI and its impact on antifungal therapy.^92^

## 3 Management of Plant Pathogenic Fungi

The most widely used chemical fungicides worldwide can be classified into 15 major groups, according to their chemical properties and modes of action (Table 6). Figure 5 presents the chemical structures of representative fungicides from the 15 major groups. These fungicides are categorized as either contact or systemic. Contact fungicides provide surface protection against pathogenic fungi and do not penetrate plant tissues. In contrast, systemic fungicides enter the plant and are distributed evenly through the vascular system, offering protection against pathogens capable of penetrating the plant.

**Figure 5:**
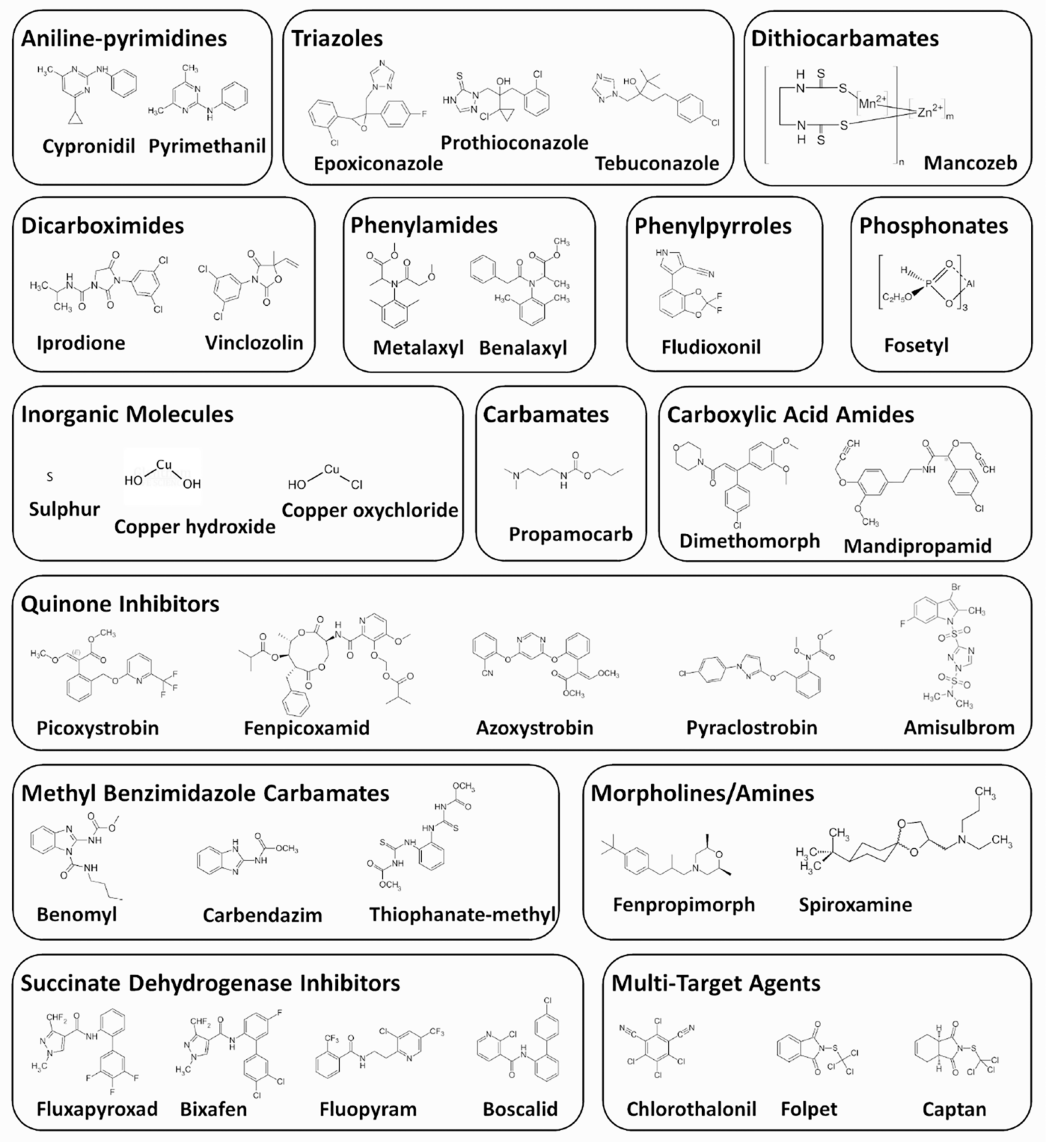
Chemical structures of representative fungicides from the 15 major groups of chemical fungicides.

**Table 6:** Most Widely Applied Chemical Fungicide Groups Globally^a^

group	chemical family	example fungicide	target	mode of action	effect
aniline pyrimidines	aniline pyrimidine	cyprodinil	cystathionine-β-synthase, cystathionine-β-lyase	inhibits secretion of hydrolytic enzymes needed for infection by interfering with methionine biosynthesis	systemic
		pyrimethanil			
demethylation inhibitors	triazole	epoxiconazole	lanosterol-14α-demethylase	inhibits ergosterol biosynthesis through lanosterol-14α-demethylase inhibition	systemic
		prothioconazole			
		tebuconazole			
dicarboximides	dicarboximide	iprodione	MAP/histidine kinase	blocks signal transduction	systemic
		vinclozolin			
dithiocarbamate (derivatives)	dithiocarbamate (derivatives)	mancozeb	multiple targets	acts on multiple targets	contact
phenylamides	acylalanine	metalaxyl (mefenoxam)	RNA polymerase I	inhibits RNA synthesis	systemic
	methylalanine	benalaxyl			
phenylpyrroles	phenylpyrrole	fludioxonil	MAP/histidine kinase	blocks signal transduction	contact
phosphonates	ethylphosphonate	fosetyl	phosphonate	inhibits mycelium growth and sporulation	systemic
inorganic molecules	inorganic sulfur	sulfur	proteins	inhibits germination, respiration, and metabolism	contact
	inorganic copper	copper hydroxide	amino acids, proteins	enzyme inhibition	
		copper oxychloride			
carbamates	carbamate	propamocarb	acetylcholinesterase	inhibits cell membrane biosynthesis by disrupting lipid biosynthesis	Systemic
carboxylic acid amides	carboxylic acid amide	dimethomorph	cellulose synthase	inhibits cell wall biosynthesis through cellulose synthase inhibition	systemic
		mandipropamid			
quinone inhibitors	enoyl ester	picoxystrobin	cytochrome *b*	inhibits cellular respiration	systemic
	carboxamide	fenpicoxamid	cytochrome bc1 complex III		
	methoxy-acrylate	azoxystrobin	cytochrome *b*		
	methoxy-carbamate	pyraclostrobin			
	sulfonamide	amisulbrom			
methylbenzimidazole carbamates	thiophanate	benomyl	β-tubulin	inhibits mitosis and cell division	systemic
		carbendazim			
		thiophanate-methyl			
morpholines/amines	pyrimidinamine	fenpropimorph	Δ8−Δ7- sterol isomerase, Δ14-sterol reductase	inhibits ergosterol biosynthesis through Δ8−Δ7- sterol isomerase/Δ14-sterol reductase inhibition	systemic
	spiroketalamine	spiroxamine	Δ14 sterol reductase		
succinate dehydrogenase inhibitors	aromatic amide	fluxapyroxad	succinate dehydrogenase/complex II	inhibits succinate dehydrogenase, blocking mitochondrial ATP production	systemic
	pyrazole carboxamide	bixafen			
	pyridyl ethylbenzamide	fluopyram			
	pyridine carboxamide	boscalid			
multitarget agents	dinitrile	chlorothalonil	multiple	acts on multiple targets	contact
	phthalimide	folpet			
		captan			

a ATP: adenosine triphosphate, MAP: mitogen-activated protein. Compiled based on tables edited by Gikas et al.^96^ and Corkley et al.^98^

Table 6 shows only a few selected examples because the number of chemical fungicides continuously increases with newer ones being brought to market, mainly in the United States.^16,93^ The opposite trend is observed in the EU, where an increasing number of fungicides are being (temporarily or permanently) withdrawn from the market and banned under the Toward Zero Pollution for Air, Water and Soil action plan and other regulations.^18,19,94^ Some withdrawn fungicides may be temporarily reauthorized by the EU in emergencies. The list of currently authorized fungicides is continuously monitored and can be found on the relevant EU web portals.

Fungi cause significant damage in the field and under storage conditions, contaminating harvested crops with mycotoxins, which are harmful secondary metabolites that pose serious health risks.^86,95^

### 3.1 Challenges in Managing Plant Pathogenic Fungi

The protection of plants against fungal pathogens continues to face new challenges in pre- and postharvest conditions.^1,96–98^ Among the most significant challenges are (1) the emergence of new fungal species in agricultural areas where they were previously unknown (due to climate change), (2) the continuous spread of fungicidal resistance, and (3) the harmful effects of chemical fungicides on the environment and human health.

#### 3.1.1 Emergence of New Plant Pathogenic Fungal Species

It has been established that due to climate change, the incidence and impact of fungal infections on plants and crops are continuously increasing in pre- and postharvest conditions.^42^ Additionally, a further significant increase is expected in the near future,^99^ resulting from direct and indirect causes. Direct causes include the emergence of plant pathogenic fungal species, such as *Alternaria solani*, *Botrytis cinerea*, *Fusarium graminearum* species complex, *Magnaporthe oryzae*, *Penicillium digitatum*, *Phytophthora infestans*, *Pythium* spp., and *Puccinia graminis* f. sp. *tritici*, in agricultural areas, where they were previously unknown. Additional direct causes include higher average temperatures, which can, for instance, shorten pathogen generation times; elevated CO_2_ levels, which either increase or decrease infection severity; and increased humidity or prolonged droughts, which enhance fungal pathogen virulence.^100^ These factors also influence the nature and extent of mycotoxin contamination under storage conditions such as the appearance of *Aspergillus flavus* and *F. graminearum* species complex.^101^ Indirect causes include reduced levels of plant resistance and changes in the rhizobiome, which may reduce the number of beneficial competitor microorganisms in the soil surrounding the roots, thereby diminishing the plant’s defense capabilities against pathogenic fungi.^100^

#### 3.1.2 Resistance

Over the past 2 decades, the widespread improper use of chemical fungicides (i.e., incorrect fungicide selection, inappropriate timing and concentration, and neglecting rainy weather periods) has caused the emergence of an increasing number of resistant plant pathogenic fungal strains.^13^ This phenomenon is primarily attributed to the genomic plasticity of fungi and their broad genetic toolkit, equipping them with excellent adaptive and resistance capabilities against fungicides.^102,103^ Figure 6 comprehensively summarizes the resistance mechanisms developed against the chemical fungicides. The underlying resistance mechanisms most commonly involve one or more of the following mechanisms: (1) target gene mutation, (2) target overproduction, (3) reduction of fungicide concentration within the cell through efflux processes, and (4) detoxification via metabolic breakdown of the fungicide.

**Figure 6:**
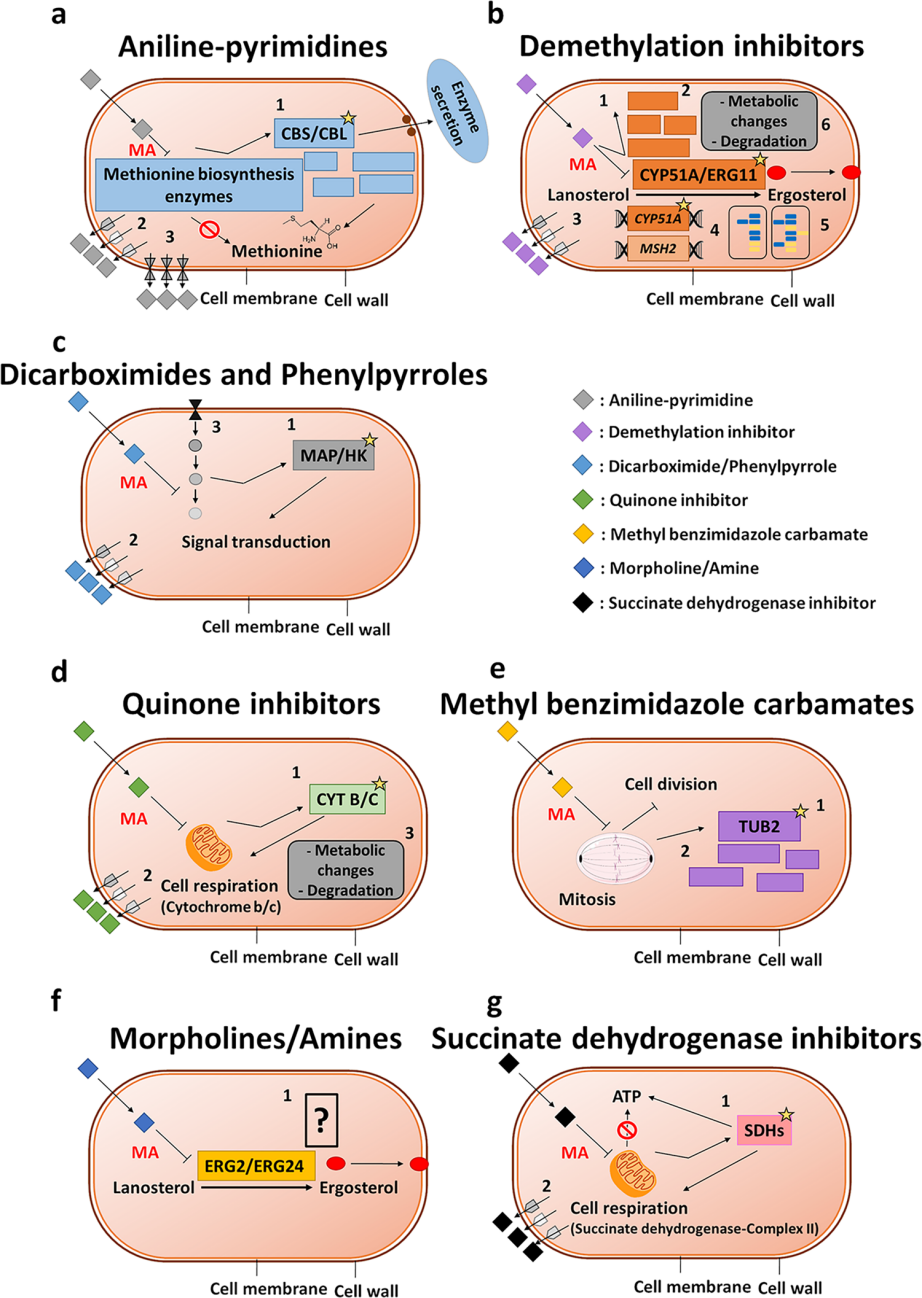
Mechanisms of action of chemical fungicide classes and the associated principal forms of acquired resistance. (a) Aniline pyrimidine resistance: (1) target gene (methionine biosynthesis enzyme genes) mutations, (2) efflux pump hyperactivity, and (3) adenosine triphosphate-binding cassette transporter overexpression. Mechanism of action (MA): inhibition of methionine biosynthesis and hydrolytic enzyme secretion. (b) Demethylation inhibitor resistance: (1) target gene (lanosterol 14α-demethylase) point mutations, (2) target overproduction, (3) efflux pump overproduction or hyperactivity, (4) hypermutation in the target and housekeeping genes, (5) heteroresistance and aneuploidy, and (6) metabolic changes and degradation. MA: lanosterol 14α-demethylase inhibition. (c) Dicarboximide and phenylpyrrole inhibitor resistance: (1) target gene (mitogen-activated protein kinase/histidine kinase) mutations, (2) efflux pump hyperactivity, and (3) adenosine triphosphate-binding cassette transporter overexpression. MA: signal transduction blocking. (d) Quinone inhibitor resistance: (1) target gene (cytochrome *b*/*c*) mutations, (2) efflux pump hyperactivity, and (3) metabolic changes and degradation. MA: cellular respiration inhibition. (e) Methylbenzimidazole carbamate resistance: (1) target gene (β-tubulin) mutations and (2) target overproduction. MA: mitosis and cell division inhibition. (f) Morpholine/amine resistance: (1) unknown. MA: Δ8−Δ7-sterol isomerase/Δ14-sterol reductase inhibition. (g) Succinate dehydrogenase inhibitor resistance: (1) target gene (succinate dehydrogenase/complex II) mutations and (2) efflux pump hyperactivity. MA: succinate dehydrogenase inhibition and mitochondrial adenosine triphosphate production blocking. CBL: cystathionine-β-lyase, CBS: cystathionine-β-synthase, CYP51A/ERG11: lanosterol 14α-demethylase, CYT B/C: cytochrome *b*/*c*, ERG2: Δ8−Δ7-sterol isomerase, ERG24: Δ14-sterol reductase, HK: histidine kinase, MAP: mitogen-activated protein kinase, *MSH2*: azole resistance gene, SDH: succinate dehydrogenase, TUB2: β-tubulin. Asterisks indicate mutations. The figure is compiled using information presented in Hawkins and Fraaije,^102^ and Brauer et al.^15^ and updated with the original literature findings.

The most common resistance mechanism against aniline pyrimidines is the overproduction of energy-dependent efflux pumps (adenosine triphosphate-binding cassette transporters), thereby reducing the intracellular concentration of the fungicide to ineffective levels. The increased activity of alternative efflux pumps and mutations in genes associated with methionine biosynthesis also contribute to resistance (Figure 6a). At least one of these resistance mechanisms has been identified in *B. cinerea*, *Oculimacula* spp., and *Venturia inaequalis*.^15^

Resistance to demethylation inhibitors is mainly due to point mutations in the gene encoding the target enzyme (i.e., mutations in lanosterol 14α-demethylase, resulting in the inability or reduced ability of azoles to inhibit enzymatic activity) or enzyme overproduction (i.e., increased enzyme production compensates for the inhibitory effect of the intracellular azole concentration). Other mechanisms contributing to resistance against demethylation inhibitors include hypermutations in both the target and housekeeping genes, heteroresistance, aneuploidy, metabolic adaptations, and fungicide degradation (Figure 6b). One or more of these resistance mechanisms have been observed in *B. cinerea*, *Brumeriela jaapii*, *Cercospora beticola*, *Monilinia fructicola*, and *P. digitatum*, *V. inaequalis*, and *Zymoseptoria tritici*.^15^

Resistance to dicarboximide and phenylpyrrole fungicides was established in *B. cinerea* and is mainly attributed to mutations in the gene encoding histidine kinase, thereby preventing the fungicide from binding to the enzyme and inhibiting its function (Figure 6c).^102^

Besides efflux pump hyperactivity, resistance to quinone inhibitors is caused by point mutations in the mitochondrial cytochrome *b* and *c* genes, preventing the fungicide from binding to its target site and inhibiting cellular respiration. These resistance mechanisms have been well characterized in *A. solani*, *Erysiphe necator*, *Pseudopernospora cubensis*, *Pyrenophora teres*, *Pythium aphanidermatum*, *Pyrenophora tritici* *-repentis*, and *V. inaequalis* (Figure 6d).^15^

To date, approximately 115 fungal species have developed resistance to methylbenzimidazole carbamates, and the mechanism has been well characterized in *B. cinerea* and *V. inaequalis*. Resistance is mainly due to point mutations in the β-tubulin gene, reducing or preventing the fungicide from binding to and inhibiting β-tubulin function (Figure 6e).^15^

Resistance to morpholine and amine fungicides has been reported in *Erysiphe* spp., *Microsphaera* spp., *Phyllactinia* spp., *Podosphaera* spp., *Sphaerotheca* spp., and *Uncinula* spp. However, the underlying molecular mechanisms remain unknown (Figure 6f).^15^

The extensive and widespread use of succinate dehydrogenase inhibitors rapidly led to the emergence of resistant fungal strains due to an amino acid substitution in histidine at position 257 of the enzyme, preventing fungicide binding. Efflux pump hyperactivity was also implicated in the resistance mechanism. Resistance to succinate dehydrogenase inhibitors has been reported in *Alternaria alternata*, *B. cinerea*, *Corynespora cassiicola*, *Didymella brioniae*, and *Podosphaera xanthii* (Figure 6g).^15^

Any discussion of fungicide resistance must address the widespread and intensive use of azole-based fungicides in agricultural areas. This significantly promotes the development of human pathogenic *A. fumigatus* strains resistant to clinically used triazoles.^45^

#### 3.1.3 Harmful Impact on the Environment and Human Health

Introducing chemical fungicides into the environment promotes the development of resistant fungal strains and directly or indirectly harms the environment and human health. Because of their physicochemical properties, many fungicides accumulate in soil and surface or groundwater, affecting water quality and exerting significant harmful effects on the organisms in these ecosystems through direct toxicity or indirect ecotoxicological processes.^96,104^

The direct toxic effects of chemical fungicides largely depend on their fungal-specific modes of action, and fungicides targeting nonfungal-specific sites may also harm other organisms. Although inorganic fungicides (e.g., copper- and sulfur-based) pose the lowest toxicological risk, they accumulate in aquatic organisms (e.g., algae, fish, and crustaceans), impact food quality upon consumption, and alter soil microbiome composition.^96^ Among chemical fungicides, demethylation inhibitors mainly harm non-plant pathogenic fungi. Although their toxicity to aquatic plants, invertebrates, and vertebrates is low, they disrupt sex steroid production in fish and amphibians, causing imbalanced gender ratios. Methylbenzimidazole carbamates exhibit low toxicity to aquatic microorganisms, plants, and vertebrates; moderate toxicity to non-plant pathogenic fungi; and low to moderate toxicity to algae, bacteria, and crustaceans. Quinone inhibitors harm aquatic invertebrates and amphibians to a low or moderate extent. Carbamates are low or moderately toxic to algae, bacteria, and crustaceans.^104^ A direct correlation exists between intensive agricultural use of fungicides and declining populations of honeybees and other critical pollinators. Some fungicides disrupt the microbiome of the digestive system of bees, resulting in metabolic imbalances and mortality.^105^

The indirect harmful effects of chemical fungicides are primarily manifested through ecotoxicological processes. Inorganic fungicides (e.g., copper- and sulfur-based) influence the quantity and quality of nutrients available to plants by altering the soil structure and microbiome composition, reducing the resistance of plants to pathogens.^96^ High levels of fungicide accumulation promote biofilm formation by pathogenic microorganisms, enhancing their resistance and virulence. Additionally, the significant toxic effects of fungicides on certain organisms disrupt predator–prey and host–pathogen balances.^105^ Mass bee mortality caused by the intensive application of fungicides causes ecological disasters and negatively impacts agricultural food production.^106^

Regarding human health, the intensive application of chemical fungicides is likely linked to various chronic illnesses (e.g., cancer and cardiovascular, respiratory, and neurological diseases). A recent extensive survey of five European countries detected the presence of at least two pesticides (including fungicides) in 84% of participants. The effects of fungicides on human health are not yet fully elucidated and warrant further investigation.^106^

The challenges discussed above may be mitigated by the previously mentioned EU action plan (Toward Zero Pollution for Air, Water and Soil),^19^ which advocates for developing novel antifungal agricultural strategies with a low environmental impact.

### 3.2 New Trends in Plant Pathogenic Fungi Management

The expectations for a new type of fungicide are similar to those discussed for antifungal drugs used in human medicine, with the most critical criteria being a broad antifungal spectrum, minimal risk of resistance development, and a fungus-specific mode of action. The first two ensure long-term and effective application, while the latter ensures safe use with minimal environmental impact.^107^ Such approaches may include developing chemical fungicides with novel modes of action, using biocontrol agents and naturally occurring biomolecules, or creating new and more effective formulations and targeted delivery methods.

#### 3.2.1 Chemical Fungicides with Novel Modes of Action

The currently available or recently under development fungicidal arsenal eliminates plant pathogenic fungi through approximately 52 different mechanisms targeting major physiological processes, such as nucleic acid metabolism, cytoskeleton and motor proteins, cell respiration, amino acid and protein synthesis, signal transduction, lipid biosynthesis, transport processes, membrane integrity, melanin synthesis, cell membrane components, and cell wall biosynthesis.^93^ Some of the components involved in these processes may be potential targets for developing new fungus-specific chemical fungicidal molecules with novel modes of action and can be identified from molecule libraries using laboratory-based or *in silico* high-throughput screening techniques. Such molecules can be specifically modified for improved fungal specificity.^15^ Chemical fungicides with novel modes of action identified and modified using these techniques include dihydroorotate dehydrogenase inhibitors, oxysterol-binding protein inhibitors, melanin biosynthesis inhibitors, and Gwt1 protein inhibitors.^108^

Dihydroorotate dehydrogenase inhibitors (e.g., ipflufenoquin and quinofumelin) bind to the enzyme to block pyrimidine-based nucleotide biosynthesis. They have demonstrated their effectiveness against *F. graminearum* and fungi, causing gray mold disease and powdery mildew, and there are no reports of cross-resistance. Olorofim, a similar antifungal molecule developed for human therapeutic use (Table 5), has demonstrated efficacy against *A. fumigatus*, *P. infestans*, and *P. aphanidermatum*.^108^

Oxysterol-binding protein inhibitors (e.g., oxathiapiprolin and fluoxapiprolin) block lipid transfer. They act on a narrow spectrum and are effective against members of the class Oomycetes. Laboratory tests suggest a moderate to high risk of resistance development and potential cross-resistance.^108^

Melanin biosynthesis inhibitors, such as tricyclazole, suppress melanin biosynthesis, which is essential for infection via the appressorium by inhibiting hydroxy-naphthalene reductase or trihydroxy-dihydronaphthalene dehydratase. However, there is a high risk of resistance and cross-resistance. Tolprocarb is another melanin biosynthesis inhibitor, but it inhibits polyketide synthase. Tolprocarb resistance has been observed but not cross-resistance. These compounds are primarily effective against *Pyricularia oryzae*, which causes rice blast.^108^

Gwt1 protein inhibitors, such as aminopyrifen, were developed as human therapeutics that prevent the incorporation of mannoprotein into the cell wall (Table 5). They are effective against both ascomycetous and basidiomycetous phytopathogenic fungi including *B. cinerea*, *Blumeria graminis* f. sp. *tritici*, *P. xanthii*, and *Puccinia recondita*. However, resistance and cross-resistance may develop.^108^

Due to the aforementioned high-throughput screening techniques, fungicidal candidate molecules with novel, previously unknown mechanisms of action have been identified, such as tebufloquin against *Pestalotia longiseta* and *P. oryzae*; picarbutrazox against *Pythium* spp., *Fusarium* spp., and *Rhizopus* spp.; and dipymetitrone against *Phytophthora* spp., *Botrytis* spp., and powdery mildew.^108^

#### 3.2.2 Biocontrol Agents and Biomolecules

The application of biocontrol agents or biomolecules derived from various organisms as pesticides has a lower environmental impact and ecological risk compared with chemical fungicides.^109^

Several fungicidal biocontrol agents have been identified in recent years. These agents are typically antagonistic microorganisms that effectively suppress or eliminate pathogens from their environment via competition, mycoparasitism, or antibiosis, without harming the host or the rhizobiome.^109^ They may also release molecules into the environment that enhance plant defense mechanisms. For soilborne plant pathogenic fungi (*Fusarium* spp., *Pythium* spp., *Phytophthora* spp., *Rhizoctonia solani*, *Sclerotinia* spp., *Sclerotium rolfsii*, and *Verticillium dahliae*), *Trichoderma* species (such as *T. atroviride*, *T. hamatum*, *T. harzianum*, and *T. viride*) are particularly effective, along with various bacterial species (*Bacillus* spp., *Burkholderia* spp., *Pseudomonas* spp., and *Streptomyces* spp.). Topically applied biocontrol agents are effective against airborne fungal pathogens, such as *Chaetomium* spp. against *Athelia bombacina* and *V. inaequalis*, *Rhodotorula kratochvilovae* against *Monilinia* spp., and *Tuberculina maxima* against *Cronartium ribicola*. Additionally, *Bacillus* spp. effectively inhibit the growth of *A. alternata*, whereas *Pseudomonas protegens* inhibits the growth of *B. cinerea*, *A. alternata*, *A. niger*, *P. expansum*, *Neofusicoccum parvum*, *Phaeomoniella chlamydospora*, and *Phaeoacremonium aleophilum*. Under storage conditions, *Pseudomonas syringae* proved to be effective against *B. cinerea* and *P. expansum*, whereas *Aureobasidium pullulans* mitigated the damage caused by *B. cinerea* and *Rhizopus stolonifer*. Currently approved biocontrol products include those based on *Bacillus* spp., *Pseudomonas chlororaphis*, *Streptomyces* spp., *Ampelomyces quisqualis*, *A. pullulans*, *Candida oleophila*, *Clonostachys rosea*, *Coniothyrium minitans*, *Pythium oligandrum*, *Saccharomyces cerevisiae*, *Trichoderma* spp., and *Verticillium albo* *-atrum*.^109^

Biomolecules derived from plants or other organisms can also effectively protect plants against fungi by destroying fungal pathogens and stimulating the defense system, growth, and biotic stress resistance of plant hosts.^110^ Examples include plant probiotics,^110^ phytochemicals,^111^ and short double-stranded RNA molecules and antifungal peptides.^112^

Plant probiotics exert a multifaceted effect, promoting plant growth (e.g., stimulating auxin, ethylene, gibberellin, and cytokinin production), making nutrients accessible (e.g., bacterial siderophores), and increasing resistance to stress factors, including biotic factors (e.g., antifungal compounds and pathogen inhibitors).^110^ Phytochemicals include raw plant extracts or secondary metabolites derived from plants. Many plant extracts have effectively mitigated fungal infections and damaged fungal pathogens. Due to their fungal selectivity, they pose a minimal risk of resistance development and environmental harm. However, challenges such as large-scale, cost-effective production, and field-effective formulations must be overcome. Currently, seven phytochemical products from plants (e.g., *Reynoutria sachalinensis*, *Swinglea glutinosa*, *Melaleuca alternifolia*, and *Thymus vulgaris*) are commercially available, mainly effective against *Botrytis*, *Fusarium*, and powdery mildew infections.^111^

Plant protection strategies based on short double-stranded RNA molecules and peptides are available at various stages of technological advancement. Double-stranded RNA molecules primarily exert their antifungal effects through RNA interference, targeting genes essential for fungal cellular structure or metabolism.^112^ Numerous antifungal peptides, originating from various organisms or synthesized *de novo*, have effectively inhibited the growth of field crop pathogens and storage pest fungi.^86,113,114^ Their advantageous properties for specific agricultural applications include fungicidal activity at relatively low concentrations, low risk of resistance development, synergistic interactions with some chemical fungicides and plant defense systems against pathogens, and potential plant growth stimulation by influencing symbiosis. However, their disadvantage is the relatively high production cost. As previously discussed, antifungal peptides and proteins inhibit the growth of plant pathogenic fungi or kill them through distinct mechanisms (e.g., cell membrane disruption, inducing apoptosis).^113,114^ Two approaches can be used to integrate antifungal peptides/proteins into plant and crop protection: (1) development of cisgenic or transgenic plants that express large amounts of self-derived or foreign antifungal peptides/proteins, thereby enhancing their resistance to fungal pathogens, and (2) applying antifungal peptides/proteins as biofungicides in spray formulations. The first approach faces significant challenges due to current regulations on genetically modified plants^115^ and consumer aversion toward foods derived from genetically modified organisms.^116^ Thaumatin­(-like) proteins, thionins, defensins, hevein-like proteins, knottin-like proteins, lipid transfer proteins, and snakin are suitable candidates for creating antifungal peptide/protein-overproducing cisgenic and transgenic plants. A practical example is an alfalfa antifungal peptide-overproducing potato plant, which showed increased resistance against *Phytophthora cactorum* and *F. solani* infections under field conditions. High production costs, poor solubility, instability under environmental conditions, and unresolved formulation and delivery technology hamper the application of surface spray formulations of antifungal peptides/proteins in fields.^112–114^ Thus, only a few antifungal peptide- and protein-based sprays have advanced beyond laboratory or greenhouse trials. For example, under field conditions, NmDef02 from *Nicotiana megalosiphon* showed efficacy against *Peronospora hyoscyami*; trichogin from *Trichoderma* against *Plasmopara viticola*; synthetic body protection compound peptides against *A. niger*, *Fusarium oxysporum*, and *P. expansum*, *R. stolonifer*, and *Stemphylium vesicarium*; and ϵ-PL against *B. cinerea*.^114^ Some antifungal peptides/proteins of various origins inhibit the proliferation of mycotoxigenic fungi (primarily *Aspergillus* spp., *Fusarium* spp., and *Penicillium* spp.) or reduce their mycotoxin production when sprayed onto crops in storage conditions by inducing oxidative stress and inhibiting specific enzymatic components of biosynthetic pathways.^86^

#### 3.2.3 Formulation, Delivery, and Nanotechnology

Issues such as safe and effective application and resistance development arising with currently used and newly introduced chemical fungicides may be overcome by using novel formulations and delivery techniques. A new formulation must meet the following requirements: environmentally friendly, resistant to extreme environmental conditions (e.g., UV radiation, pH, temperature, evaporation, oxidation, etc.), favorable physicochemical properties for targeted delivery, controllable and precise application, and efficacy at low concentrations. Currently, nanotechnology, which involves encapsulating existing chemical fungicides or biomolecules (e.g., dsRNA, peptides, and plant metabolites) in nanoparticles or binding them to nanoparticles,^117,118^ or the utilization of specific nanoparticles as pesticides, provides an excellent solution to these challenges.^15^

Experiments have shown that nanoparticle use reduces the environmental impact and risk of resistance development, makes fungicides more resistant to extreme environmental conditions, enables faster and more efficient delivery, and is effective at lower doses than previously used. However, the disadvantages of nanofungicides, such as complex production technologies, high costs, and unclear effects on human health, hinder their application.^117,118^ Nanomaterials used as fungicide carriers and packaging include carbon-based materials (e.g., graphene and graphene oxide), synthetic anionic substances (e.g., layered double hydroxides), silica nanoparticles, various polymers, and metal nanoparticles.^118^ Iron­(II) oxide, copper oxide, and titanium dioxide may be considered as stand-alone nanofungicides.^15^ Many nanofungicides are under development, but to date, none have been approved for agricultural use in the EU due to the unclear long-term effects on animal and human health.^119^

## 4 Conclusions and Outlook

Despite the efforts of decision-making bodies and the promising solutions discussed, fungal infections continue to show a concerning and rising trend. Although accelerating the implementation of the action plans outlined in the WHO fungal priority pathogens list may provide an effective solution for mitigating this issue,^3^ it requires significantly more financial support and heightened public attention. The chronic underfunding of mycological research must be addressed,^120^ with support and substantial investment directed toward studies focused on improving human welfare in the areas of healthcare, food supply, and food security. Public awareness of global fungal-related challenges must also be raised, urging communities to unite to address these problems. A potential approach is the introduction of new initiatives involving civilian participation in research efforts, such as sample collection for antifungal drug resistance surveys,^121,122^ monitoring the spread of fungicide resistance,^123^ or even engaging farmers in testing new agricultural antifungal strategies in the field.^107^ Increased public attention and societal collaboration are expected to influence local political leaders and decision-making bodies, driving antifungal problem-solving at the regional level, eventually expanding to global-scale solutions.

## Abbreviations

AI: artificial intelligence
Als3: agglutinin-like sequences
COVID-19, EU: coronavirus disease 2019; European Union
Gwt1: cell wall transfer protein 1
Hsp90: heat shock protein 90
WHO: World Health Organization
